# Kaempferol-Driven Inhibition of Listeriolysin O Pore Formation and Inflammation Suppresses Listeria monocytogenes Infection

**DOI:** 10.1128/spectrum.01810-22

**Published:** 2022-07-20

**Authors:** Tingting Wang, Baichen Liu, Can Zhang, Tianqi Fang, Ying Ding, Ge Song, Siqi Zhang, Linlin Shi, Xuming Deng, Jianfeng Wang

**Affiliations:** a State Key Laboratory for Zoonotic Diseases, Key Laboratory of Zoonosis Research, Ministry of Education, Institute of Zoonosis, College of Veterinary Medicine, Jilin University, Changchun, Jilin, China; b The Second Bethune Clinical Medical College of Jilin University, Changchun, China; University of Greifswald

**Keywords:** *Listeria monocytogenes*, anti-infection, anti-inflammation, kaempferol, listeriolysin O (LLO) inhibitor

## Abstract

Listeria monocytogenes remains a nonnegligible cause of foodborne infection, posing a critical threat to public health. Under the global antibiotic crisis, novel alternative approaches are urgently needed. The indispensable role of listeriolysin O (LLO) in the intracellular life cycle, barrier penetration, colonization, and systemic dissemination of L. monocytogenes renders it a potent drug target, which means curbing L. monocytogenes via interfering with LLO-associated pathogenic mechanisms. Here, we identified kaempferol, a natural small molecule compound, as an effective LLO inhibitor that engaged the residues Glu437, Ile468, and Tyr469 of LLO, thereby suppressing LLO-mediated membrane perforation and barrier disruption. Moreover, we found that kaempferol also suppressed host-derived inflammation in a distinct way independent of LLO inhibition. The *in vivo* study revealed that kaempferol treatment significantly reduced bacterial burden and cytokine burst in target organs, thereby effectively protecting mice from systemic L. monocytogenes infection. Our findings present kaempferol as a potential therapeutic application for L. monocytogenes infection, which is less likely to induce drug resistance than antibiotics because of its superiority of interfering with the pathogenesis process rather than exerting pressure on bacterial viability.

**IMPORTANCE** Currently, we are facing a global crisis of antibiotic resistance, and novel alternative approaches are urgently needed to curb L. monocytogenes infection. Our study demonstrated that kaempferol alleviated L. monocytogenes infection via suppressing LLO pore formation and inflammation response, which might represent a novel antimicrobial-independent strategy to curb listeriosis.

## INTRODUCTION

Listeria monocytogenes is an opportunistic pathogen responsible for listeriosis, a sporadic disease associated with the consumption of highly contaminated food, especially ready-to-eat food products. In China, the foodborne proportion of L. monocytogenes is up to 99% (1). L. monocytogenes can rapidly replicate in spoiled food products because of the increased alkalinity (2). In addition, the microorganism can survive at refrigeration temperatures and high salt concentrations; thus, it is ubiquitous in water, soil, refrigerators, and the food processing industry, as well as silage and livestock environments (3), posing a serious threat to public health. Although the cases of L. monocytogenes infection per year are much lower than those of other foodborne illnesses (about 23,150 cases worldwide), the pathogen stands out for its high mortality among infected patients (20 to ~30%) (4). For most normal adults who are healthy and immunocompetent without specific medical conditions or pregnancy, the ingestion of severely contaminated food commonly leads to mild to severe gastroenteritis symptoms that may resolve spontaneously. However, equal or even lower levels of food contamination can cause systematic clinical syndromes with high risk of death in aging populations, newborns, and immunocompromised persons, most frequently meningitis and sepsis. In particular, the infection of pregnant individuals can cause abortion and other complications related to pregnancy (5). In ruminants, L. monocytogenes can cause invasive infection in fetuses and the central and nervous system, resulting in abortion and circling disease, respectively (6).

The combination of ampicillin or amoxicillin with the aminoglycoside antibiotic gentamicin is the standard clinical therapy for severe listeriosis (2). In nonmeningeal infections, vancomycin is the best choice, while erythromycin is more applicable for pregnant women. Compared with other resistant bacteria such as Staphylococcus aureus, Acinetobacter baumannii, and Enterococcus faecalis, L. monocytogenes retains a higher rate of susceptibility to broad-spectrum antibiotics at present. However, the efficiency of antibiotics against L. monocytogenes is also declining owing to the frequent emergence of resistant strains that acquire resistance gene from other donor organisms (7). Notably, commensal microbes in the host intestine provide the first-line defense against L. monocytogenes infection via occupying the intestinal surface, producing diverse products with antimicrobial activity, consuming nutrients, or modulating immune defense pathways (8, 9). Thus, the side effect of antibiotics on the host microbiota may likely facilitate the expansion and traversing of L. monocytogenes in the intestine and therefore lead to treatment failure, especially in immunocompromised individuals and in cases with intestinal disease. Given the global crisis of antibiotic resistance that we are facing and the limitations of antibiotic therapy for L. monocytogenes infection, continuous studies of other alternative therapeutic strategies are needed.

The successful adaptation of L. monocytogenes to diverse mammals and birds relies on the evolutions of the interaction between relevant virulence determinants and host defense systems. Among these virulence factors, the cholesterol-dependent pore-forming toxin listeriolysin O (LLO) plays a central role in L. monocytogenes pathogenicity. Unlike other cholesterol-dependent cytolysins (CDCs) that act like a bazooka to stimulate lytic cell death and tissue damage (10), LLO has been characterized as a phagosome-specific lysin owing to its optional activity under low-acid conditions (pH < 6) of mature phagosome and its decisive contribution to phagosome escape of L. monocytogenes into cytoplasm (11). Intriguingly, accumulating evidence reveals that LLO is more likely to function like a Swiss army knife, because it is not limited to allowing intracellular replication and spreading, the unique CDC toxin also has many other potential activities that depend not on large pores but, rather, on small membrane perforations caused by incomplete pores (12, 13). LLO is most known for its function in phagosome escape by perforating the phagosomal membrane (14). Interestingly, LLO secreted by L. monocytogenes within the phagosome also hinders lysosome killing of bacteria through suppressing reactive oxygen species (ROS) production (15). In addition, LLO pore formation in the cytosol of host cells would promote L. monocytogenes infection via causing SUMOylation-associated protein degradation (16). Importantly, similar to other CDCs, the extracellular LLO also perforates the host cell membrane and therefore causes intricate cellular events that benefit L. monocytogenes infection. For example, LLO has been reported to promote L. monocytogenes internalization before cell entry through activating a Ca^2+^-dependent cPKC/Rac1/Arp2/3 signaling pathway (17). Extracellular LLO can also evoke an inflammatory response by perturbing ionic homeostasis, especially K^+^ and Ca^2+^ (18, 19). Notably, the alteration of Ca^2+^ homeostasis subsequent to LLO pore formation would favor infection via remodeling the mitochondrial network and endoplasmic reticulum (ER) response. Surprisingly, it has been proposed that the host cell signaling activated by the pore formation of extracellular LLO has a pronounced impact on subsequent vacuolar escape of L. monocytogenes, which might be related to the effects of ionic homeostasis on the endosomal network (20). The multifaceted effects of LLO pore formation on the host cell highlight its extraordinary contribution to L. monocytogenes pathogenesis; thus, the toxin represents a promising drug target for the treatment of L. monocytogenes infection.

Given the great importance of LLO in the pathogenesis of L. monocytogenes, we performed LLO-targeted biochemical screening and identified kaempferol, a type of flavanol present in many plants and some dietary sources, as an effective blocker of LLO. Further study revealed that kaempferol specifically inhibited LLO pore formation by engaging the residues Glu437, Ile468, and Tyr469 of LLO but did not exhibit a detectable effect on bacterial viability and toxin production, indicating that the natural compound may be easily induce drug resistance compared to traditional antibiotics. LLO-mediated cytotoxicity and barrier dysfunction were both significantly repressed by kaempferol. Additionally, we found that kaempferol suppressed L. monocytogenes-evoked inflammation mainly through inhibiting MyD88-dependent inflammation signaling. In a murine systemic infection model, kaempferol treatment effectively protected mice from L. monocytogenes infection, with reduced bacterial burden and cytokine levels in the main target organs of liver and spleen, therefore alleviating pathological damage and prolonging survival time. These results characterize kaempferol as an effective LLO inhibitor and would render this natural small molecule an alternative lead compound to curb L. monocytogenes infection.

## RESULTS

### Kaempferol exerted effective suppression of LLO pore formation.

**FIG 1:**
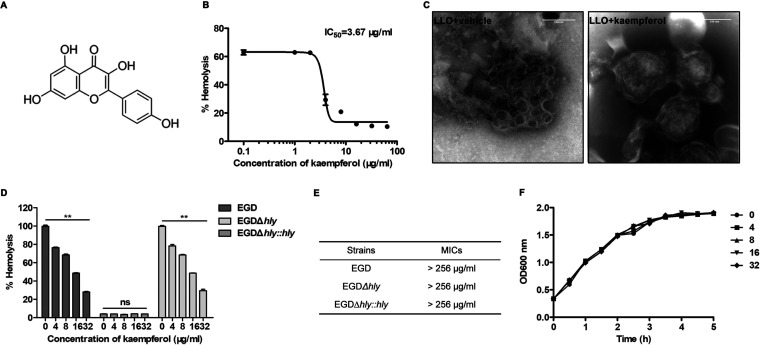
Hemolysis-based screening identifies kaempferol as an effective inhibitor of LLO pore formation. (A) Chemical structure of kaempferol. (B) Dose response curve of kaempferol in the hemolysis assay. Recombinant LLO (0.1 μg/mL) pretreated with DMSO or indicated concentrations of kaempferol for 30 min was incubated with freshly washed sheep erythrocytes for 20 min. Hemoglobin release was measured on a Tecan microplate reader at 543 nm absorbance. The absorbance of samples treated with 0.1% Triton X-100 was regarded as the reference value for 100% lysis activity. Hemolysis was defined as the value of each sample relative to the Triton X-100-treated sample. (C) Negative-stain electron microscopy images of sheep erythrocyte ghosts that were incubated with LLO in the presence of DMSO vehicle or 32 μg/mL kaempferol. Arrows point to LLO pores. Scale bar = 100 nm. (D) Kaempferol suppressed the hemolytic activity of LLO naturally produced from L. monocytogenes. Culture supernatants from wild-type L. monocytogenes strain EGD, its *hly*-deficient mutant EGDΔ*hly*, and the complementation strain EGDΔ*hly*::*hly* were harvested and incubated with the indicated concentrations of kaempferol for the hemolysis assay. (E) The MIC values of kaempferol for L. monocytogenes strains used in the study. (F) The growth curves of L. monocytogenes EGD in the presence of the indicated concentrations of kaempferol. Similar results were obtained from three independent experiments. Data are presented as means ± SEM (*n* = 3). **, *P *< 0.01 compared to the vehicle-treated group.

Acting like a Swiss army knife, LLO plays an indispensable role in L. monocytogenes infection, and most contributions of LLO in L. monocytogenes pathogenicity are dependent on its pore-forming activity. Based on this fact, we performed hemolysis-based screening to discover effective inhibitors of LLO pore formation. Kaempferol (Fig. 1A), a bioactive flavonoid broadly present in dietary sources and traditional Chinese herbs, exhibited a potent suppressive effect on the hemolytic activity of LLO, which had a 50% inhibitory concentration (IC_50_) of 3.67 ± 0.09 μg/mL (Fig. 1B).

The reduced hemolysis of LLO was a direct result of hindered formation of oligomer pores on the cell membrane; therefore, we further examined the structures of LLO on the membranes of sheep erythrocyte ghosts. Consistently, LLO protein formed large rings on the membranes, with diameters of 20 to 50 nm (Fig. 1C); some incomplete rings or arcs were also observed at the edges of membranes. In contrast, the addition of 32 μg/mL kaempferol almost completely blunted the formation of LLO rings, indicating that kaempferol blocked LLO pore formation and subsequently repressed its hemolytic activity.

Next, we evaluated the effect of kaempferol on the hemolytic activity of native LLO produced by L. monocytogenes. As expected, the hemolysis of the culture supernatants harvested from both wild-type L. monocytogenes EGD and the complementation strain EGD*Δhly*::*hly* was dose-dependently inhibited by kaempferol (Fig. 1D). However, kaempferol treatment did not affect the hemolytic activity of the culture supernatants from EGD*Δhly*, which hardly caused hemolysis (Fig. 1D), suggesting that kaempferol inhibited the pore-forming activity of native LLO derived from L. monocytogenes.

To investigate whether kaempferol exerted antimicrobial activity, the MICs and growth curves of L. monocytogenes were determined at the indicated concentrations. As shown in Fig. 1E, the MICs of kaempferol for L. monocytogenes strains used in this study were no less than 256 μg/mL. Moreover, no visible inhibitory effect on bacterial viability was observed at each time point under the concentrations required for hemolysis inhibition (Fig. 1F).

Collectively, these data demonstrated that the small molecule kaempferol represents a potential candidate for L. monocytogenes infection via effectively suppressing LLO pore formation rather than bacterial viability.

### Kaempferol attenuated L. monocytogenes-caused cytotoxicity via targeting LLO.

**FIG 2:**
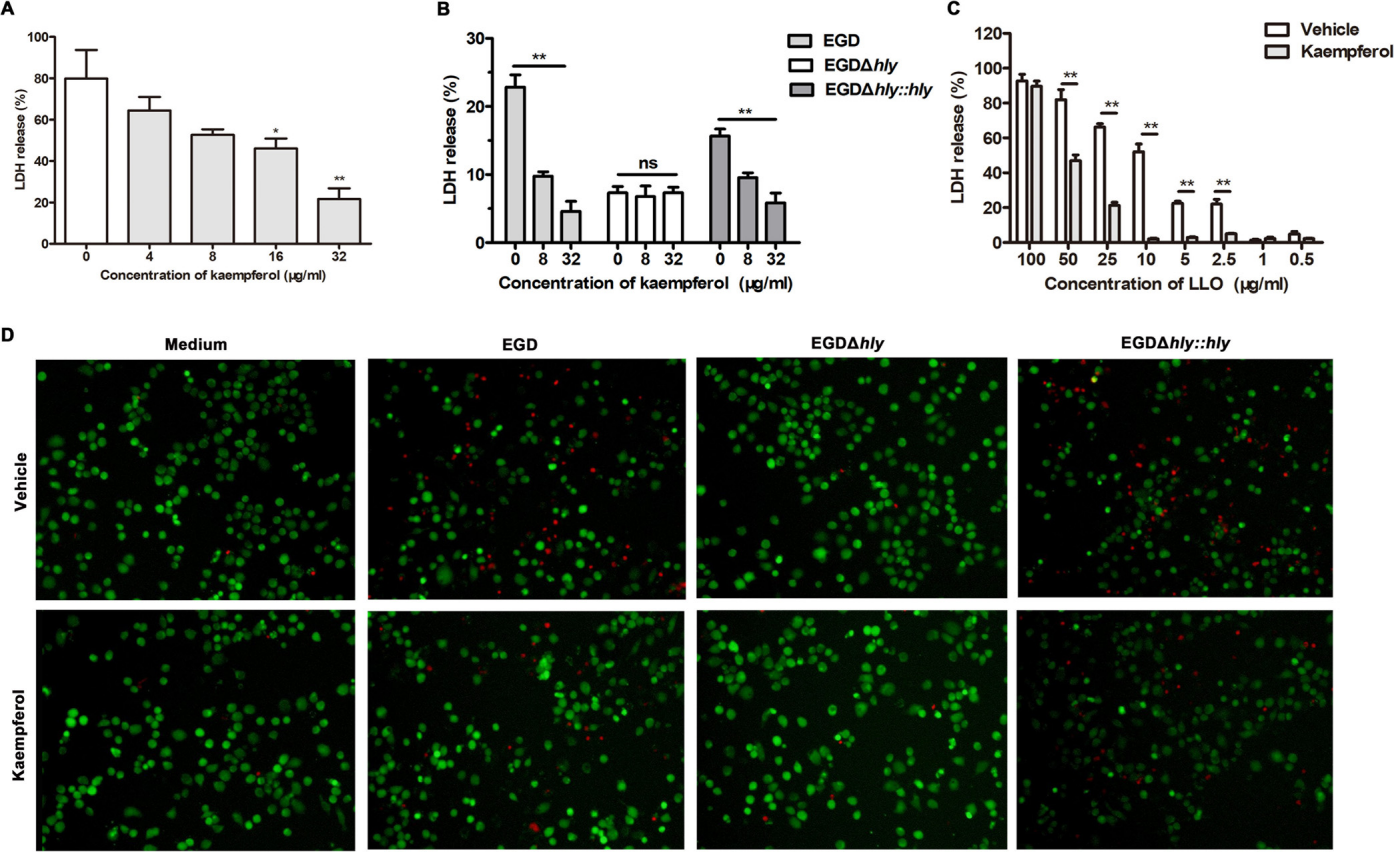
Kaempferol reduces L. monocytogenes-caused cytotoxicity via targeting LLO. (A) Cytotoxicity in J774 A.1 cells infected with wild-type L. monocytogenes strain EGD MOI20 for 6 h in the presence of various concentrations of kaempferol. (B) Cytotoxicity in J774 A.1 cells infected with wild-type L. monocytogenes strain EGD, its *hly*-deficient mutant EGDΔ*hly*, and the complementation strain EGDΔ*hly*::*hly* (MOI, 50) for 6 h in the presence of the indicated concentrations of kaempferol. (C) Cytotoxicity in Caco-2 cells stimulated by various doses of LLO in the presence of DMSO vehicle or 32 μg/mL kaempferol. (D) Live/Dead staining of J774 A.1 cells infected with wild-type L. monocytogenes strain EGD, its *hly*-deficient mutant EGDΔ*hly*, and the complementation strain EGDΔ*hly*::*hly* (MOI, 50) for 6 h in the presence of DMSO vehicle or 32 μg/mL kaempferol. Similar results were obtained from three independent experiments. Data are presented as means ± SEM (*n* = 3). **, *P* < 0.01 compared to the vehicle-treated group.

The most essential function of LLO has been considered to mediate intracellular survival of L. monocytogenes, which is indeed critical for L. monocytogenes pathogenesis. However, accumulating evidence supports the idea that, as an extracellular pore-forming toxin, LLO also perforates the host cell membrane and thus causes appreciable cell death independent of host cell invasion (21–23). Using the J774A.1 cell line, we found that challenge of wild-type L. monocytogenes EGD caused obvious cytotoxicity, while kaempferol dose-dependently reduced the cytotoxicity (Fig. 2A). Further study revealed that the suppressive effect was dependent on LLO, because the lack of *hly* led to the invalidation of kaempferol, while the complementary of *hly* restored above observations (Fig. 2B). Consistently, LLO-induced cytotoxicity in Caco-2 cells was also remarkably reduced by 32 μg/mL kaempferol, even at an extremely high dose of LLO (50 μg/mL) (Fig. 2C). Live/Dead staining revealed that both wild-type L. monocytogenes EGD and the *hly-*complementary strain EGDΔ*hly*::*hly* induced membrane-damaged cell death, where the dead cells were stained with membrane-impermeable red fluorescent dye. Conversely, no visible cell death was observed in cells infected with the *hly-*deficient strain EGDΔ*hly* or without any stimulation or in the cells exposed to EGD with the addition of 32 μg/mL kaempferol (Fig. 2D). Taken together, these results suggested that kaempferol effectively protected cells from L. monocytogenes-caused cytotoxicity by targeting LLO pore formation.

### Kaempferol suppressed the L. monocytogenes*-*evoked inflammation response.

**FIG 3:**
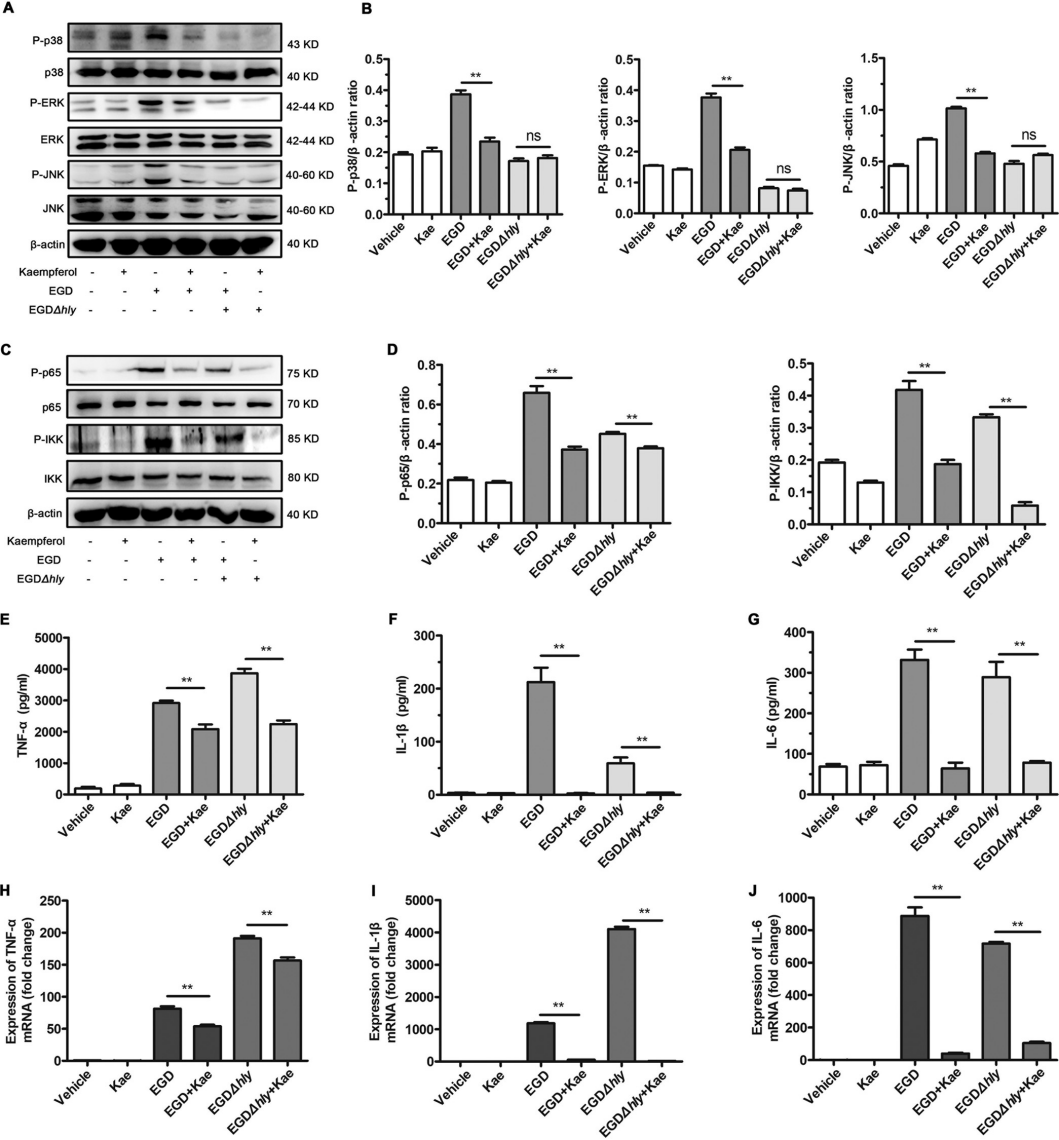
Kaempferol suppresses the L. monocytogenes-evoked inflammation response. (A) Western blot analysis of MAPK signaling cascades in MPMs infected with wildtype L. monocytogenes strain EGD and its hly-deficient mutant EGDDhly (MOI, 5) for 5 h in the presence of DMSO vehicle or 32 mg/mL kaempferol. (B) The levels of P-p38, P-ERK and P-JNK relative to β-actin determined using densitometry are shown. (C) Western blot analysis of NF-κB signaling cascades in MPMs infected with wild-type L. monocytogenes strain EGD and its hly-deficient mutant EGDDhly (MOI, 5) for 5 h in the presence of DMSO vehicle or 32 mg/mL kaempferol. (D) The levels of P-p65 and P-IKK relative to β-actin determined using densitometry are shown. (E to G) The levels of TNF-α (E), IL-1β (F), and IL-6 (G) in the coculture supernatants were detected using ELISA. (H to J) The transcriptions of TNF-α (H), IL-1β (I), and IL-6 (J) were determined by RT-qPCR. All the genes were normalized to the housekeeping gene GAPDH. Similar results were obtained from three independent experiments. Data are presented as means 6 SEM (*n* = 3). **, *P*, 0.01 compared to the vehicle-treated group.

LLO also manipulates the host inflammation response by causing ion oscillations associated with membrane perforation (24). Studies of mouse peritoneal macrophages (MPMs) verified that the wild-type L. monocytogenes EGD infection caused pronounced activation of mitogen-activated protein kinase (MAPK) and NF-κB pathways (Fig. 3A to D), with a significant increase in the phosphorylation levels of p38, extracellular signal-regulated kinase (ERK), Jun N-terminal protein kinase (JNK), p65, and IκB kinase (IKK), while 32 μg/mL kaempferol treatment significantly hindered the activation of these signaling. Although the activation of MAPK and NF-κB pathways was much lower in cells infected by EGDΔ*hly*, the slight activation NF-κB p65 and IKK were both significantly blunted by kaempferol (Fig. 3A to D), indicating that the anti-inflammation activity of kaempferol upon L. monocytogenes was not entirely dependent on LLO inhibition but that an LLO-independent mechanism was also involved. Accordingly, the secretion and transcription of the inflammation cytokines tumor necrosis factor alpha (TNF-α) (Fig. 3E and H), interleukin-1β (IL-1β) (Fig. 3F and I), and IL-6 (Fig. 3G and J) were both suppressed by kaempferol in cells infected by EGD and EGDΔ*hly*, confirming the LLO-independent anti-inflammation mechanism of kaempferol for L. monocytogenes infection.

**FIG 4:**
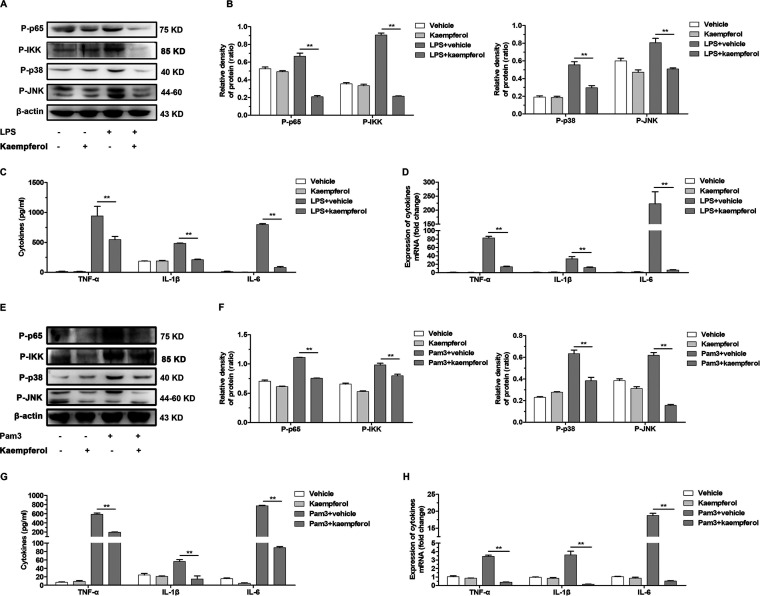
Kaempferol suppresses the MyD88-dependent inflammatory response downstream of TLR2/4. (A) Western blot analysis of NF-κB and MAPK signaling cascades in MPMs stimulated with TLR4 ligand LPS (1 mg/mL) in the presence of DMSO or 32 mg/mL kaempferol. (B) The levels of P-p65, P-IKK, P-p38 and P-JNK relative to β-actin determined using densitometry are shown. (C) The levels of cytokines TNF-α, IL-1β, and IL-6 in the culture supernatants were detected using ELISA. (D) The transcriptions of these proinflammatory genes were determined by RT-qPCR. Notably, all the genes were normalized to the housekeeping gene GAPDH. (E) Western blot analysis of NF-κB and MAPK signaling cascades in MPMs stimulated with the TLR2 ligand Pam3CSK4 (1 mg/mL) in the presence of DMSO or 32 mg/mL kaempferol. (F) The levels of P-p65, P-IKK, P-p38 and P-JNK relative to β-actin determined using densitometry are shown. (G) The levels of cytokines TNF-α, IL-1β, and IL-6 in the culture supernatants were detected using ELISA. (H) The transcriptions of these proinflammatory genes were determined by RT-qPCR. Notably, all thegenes were normalized to the housekeeping gene GAPDH. Similar results were obtained from three independent experiments. Data are presented as means 6 SEM (*n* = 3). **, *P*, 0.01 compared to the vehicle-treated group.

To interrogate the potential mechanism by which kaempferol suppressed L. monocytogenes-evoked inflammation independent of LLO, we further enumerated the effect of kaempferol on the inflammatory response induced by Toll-like receptor 4 (TLR4) and TLR2 ligands. The results revealed that kaempferol remarkably repressed MyD88-dependent activation of NF-κB and MAPK signaling responses to TLR4 ligand lipopolysaccharide (LPS) (Fig. 4A and B), which was consistent with previous studies (25, 26). Consequently, LPS-induced production of inflammatory cytokines was also significantly attenuated by kaempferol treatment (Fig. 4C and D). Importantly, similar suppression was also observed in the TLR2-mediated inflammation response, where the inflammation signaling and the burst of proinflammatory gene response to TLR2 ligand Pam3CSK4 were almost all blocked by 32 μg/mL kaempferol (Fig. 4E to H). These data further implicated a potential role of kaempferol in MyD88-dependent activation of inflammation signaling downstream of TLRs.

Taken together, these results confirmed that kaempferol could effectively suppress the L. monocytogenes-caused inflammation response through eliminating LLO perforation and suppressing the MyD88-dependent inflammation response upon recognition of L. monocytogenes by pattern recognition receptors.

### Kaempferol hindered LLO-induced barrier disruption of the Caco-2 monolayer.

**FIG 5:**
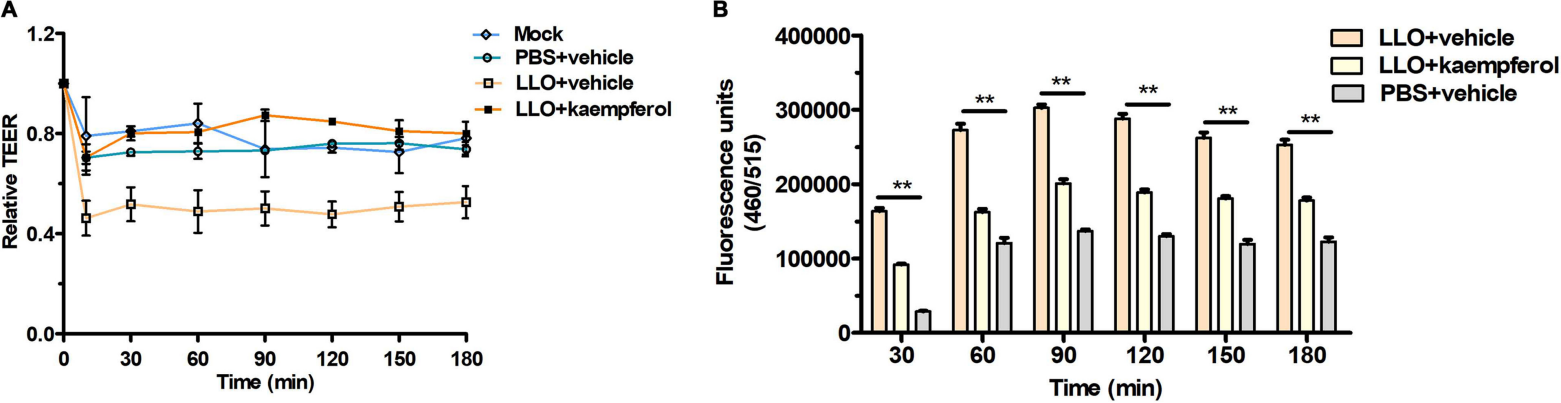
Kaempferol preserves barrier integrity of the Caco-2 monolayer upon LLO stimulation. (A) Relative changes of transepithelial electrical resistance (TEER) following the addition of LLO or PBS in the presence of DMSO vehicle or 32 μg/mL kaempferol. TEER was evaluated at an intervals of 30 min using an epithelial voltohmmeter (Millicell ERS-2), and the data are presented as the mean percentage of the initial values. (B) Permeability of the Caco-2 barrier was assessed by quantifying the percolated fluorescein in the basal chamber. The fluorescence intensity was measured using a Tecan microplate reader with excitation at 460 nm and emission at 520 nm. Similar results were obtained from three independent experiments. Data are presented as means ± SEM (*n* = 3). **, *P* < 0.01 compared to the vehicle-treated group.

As a foodborne pathogen, the natural route of infection of L. monocytogenes is through the gastrointestinal tract, followed by systemic dissemination. Functioning as a trigger for the disruption of tight junctions, the pore formation of extracellular LLO also promotes bacterial entry and spread via compromising barrier integrity. To examine the effect of kaempferol on LLO-induced barrier disruption, we evaluated the integrity of the Caco-2 monolayer, which mimicked the intestinal epithelium barrier *in vitro*. In line with a previous study (27), LLO treatment caused a dramatic decline in transepithelial electrical resistance (TEER) within the first 10 min (Fig. 5A), along with a significant increase in transcellular permeability to sodium fluorescein (Fig. 5B). Promisingly, 32 μg/mL kaempferol effectively prevented LLO-caused loss of epithelial barrier integrity. These data demonstrated that kaempferol could effectively restrain LLO-induced barrier disruption and suggested that kaempferol might prevent the translocation of L. monocytogenes from the intestines into the to the deep organs.

### Kaempferol was protective against L. monocytogenes infection in mice.

**FIG 6:**
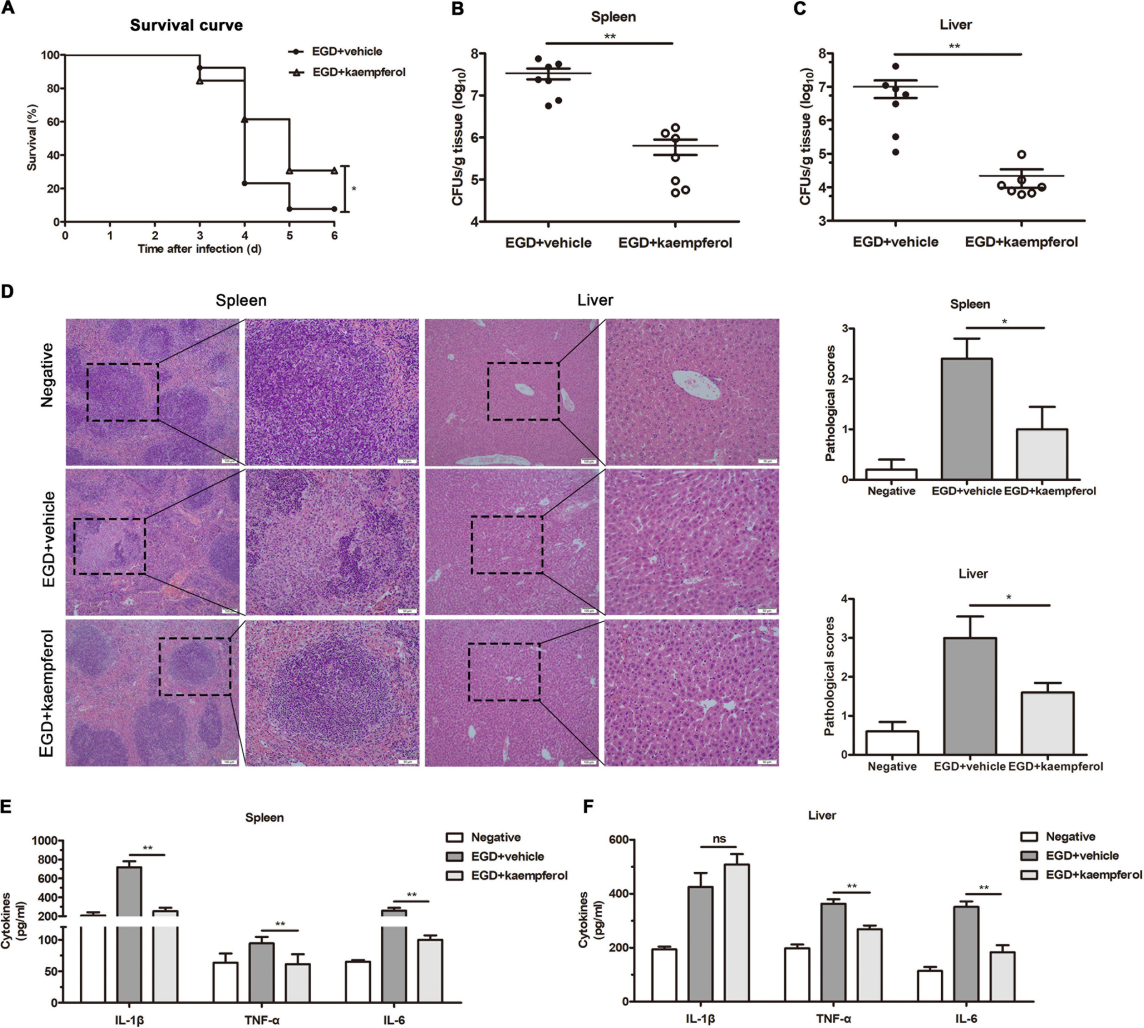
Kaempferol treatment protects mice from L. monocytogenes infection. (A) Mortality curves of mice treated with vehicle or 100 mg/kg kaempferol following intraperitoneal inoculation of 5 × 10^6^ wild-type L. monocytogenes strain EGD (*n* = 13 mice each group). Statistical analysis was performed using the log-rank (Mantel-Cox) test. (B and C) The bacterial loads in spleens (B) and livers (C) of mice that were challenged with 5 × 10^6^ EGD and administered with vehicle or 100 mg/kg kaempferol were determined by microbiological plating at 48 h postinfection (*n* = 7 mice in each group). (D) Histopathologic examination of mouse spleen and liver by H&E staining. Spleen and liver tissues from EGD infection mice with vehicle or 100 mg/kg kaempferol treatment were harvested and fixed with 10% formalin at 48 h postinfection. Scale bars = 50 μm and 100 μm. Cytokines (IL-1β, TNF-α, and IL-6) in the homogenates of spleens (E) and livers (F) were measured by ELISA (*n* = 5). Similar results were obtained from three independent experiments. Data are presented as means ± SEM. **, *P* < 0.01 compared to the vehicle-treated group.

To investigate whether kaempferol conferred effective protection against L. monocytogenes infection *in vivo*, we established a systemic infection model in BALB/c mice. Following intraperitoneal inoculation with 5 × 10^6^ EGD, approximately 92.31% of the mice died within 6 days, but 30.77% of the mice that were administered 100 mg/kg kaempferol survived (*P* = 0.0343) (Fig. 6A). Compared to the infected mice that received vehicle treatment, kaempferol-treated mice had a significantly lower bacterial load in the major target organs of spleen and liver (Fig. 6B and C), indicating limited bacterial dissemination in kaempferol-treated mice. Additionally, the good therapeutic efficiency of kaempferol was also validated by histological examination of spleen and liver using hematoxylin and eosin (H&E) staining. EGD infection caused significant histopathological injury in the spleens of vehicle-treated mice, manifested by visible lymphocyte depletion as well as obvious necrosis and congestion in the germinal center. Meanwhile, severe cell degeneration, apparent spotty necrosis, and significant inflammatory cell accumulation were observed in the liver. As expected, kaempferol-treated mice displayed slight pathological changes in these two target organs (Fig. 6D). Simultaneously, the levels of the proinflammatory cytokines IL-1β, TNF-α, and IL-6 in the spleens of vehicle-treated mice were remarkably higher than those of kaempferol-treated mice (Fig. 6E). In contrast, kaempferol treatment did not significantly reduce the IL-1β level in the liver (Fig. 6F), but the amounts of TNF-α and IL-6 were significantly diminished. Taken together, our results demonstrated that kaempferol effectively protected mice from systemic L. monocytogenes infection.

### Kaempferol engaged Glu437, Ile468, and Tyr469 on LLO to inactivate its biological activity.

**FIG 7:**
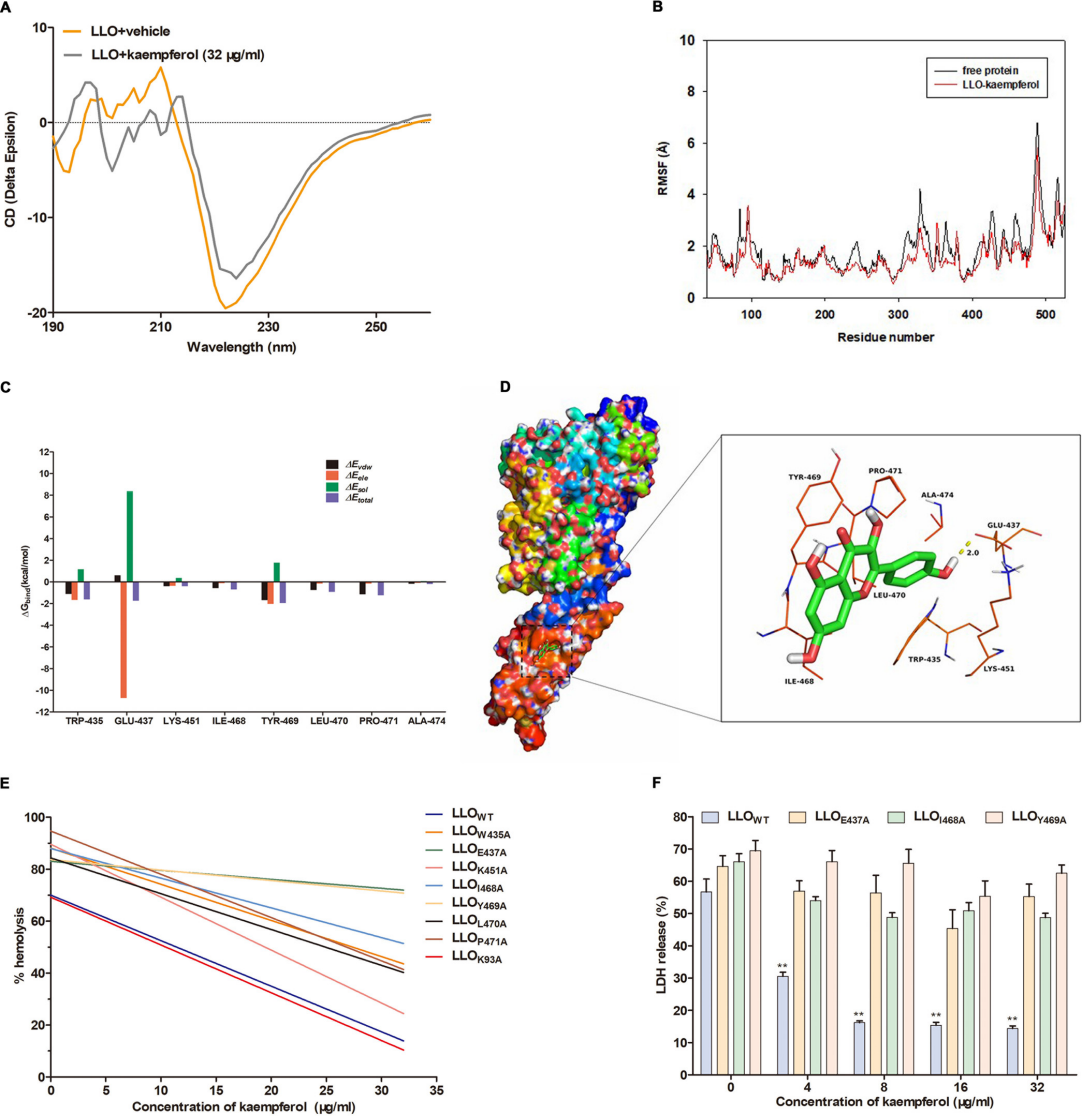
Molecular analysis of the interaction between kaempferol and LLO. (A) The circular dichroism (CD) spectra of LLO treated with DMSO or 32 μg/mL kaempferol. (B) The root-mean-square fluctuations (RMSF) of residues of the whole protein in free LLO protein and kaempferol-LLO complex based on 40-ns molecular dynamics simulations. (C) Decomposition of the binding energy on a per-residue basis in kaempferol-LLO complex. (D) The view of binding mode between kaempferol and LLO obtained from molecular docking simulations. (E) Effects of mutations in predicted binding sites on kaempferol-driven hemolysis inhibition of LLO. Recombinant LLO_WT_ and its mutants LLO_W435A_, LLO_E437A_, LLO_K451A_, LLO_I468A_, LLO_Y469A_, LLO_L470A_, LLO_P471A_, and LLO_K93A_ were pretreated with DMSO or the indicated concentrations of kaempferol for 30 min and then incubated with freshly washed sheep erythrocytes for 20 min. The hemolysis was determined as described previously. (F) Cytotoxicity in Caco-2 cells stimulated with LLO_WT_, LLO_E437A_, LLO_I468A_, and LLO_Y469A_ in the presence of the indicated concentrations of kaempferol. Similar results were obtained from three independent experiments. Data are presented as means ± SEM (*n* = 3). **, *P* < 0.01 compared to the vehicle-treated group.

To further investigate the detailed molecular mechanism of kaempferol-driven LLO inhibition, we first analyzed the secondary structure of LLO in the presence or absence of kaempferol using circular dichroism (CD). As shown in Fig. 7A, kaempferol treatment caused significant changes in CD spectra, indicating that kaempferol might inactivate LLO via altering the secondary structure of LLO protein, which would depend on its interaction with certain sites in LLO.

Then, the key residues of LLO responsible for the binding with kaempferol were identified using molecular docking analysis. The root-mean-square fluctuations (RMSF) of the residues of the whole protein in the LLO-kaempferol complex and in the free LLO clearly depicted different flexibilities in the binding site of LLO in the presence and absence of the kaempferol (Fig. 7B). The majority of the residues in the LLO binding site for kaempferol showed lower flexibility, with an RMSF of less than 3 Å compared with the free LLO, indicating that these residues appeared to be the potential binding sites to kaempferol (Fig. 7B). To gain more information about the residues surrounding the binding site and their contribution to the system, the electrostatic, Van der Waals, solvation, and total contribution of the residues to the binding free energy were calculated. In the LLO-kaempferol complex, the residue Glu-437 had a prominent electrostatic (Δ*E_ele_*) contribution, with a value of ≤10.5 kcal/mol (Fig. 7C). Detailed analysis showed that the residue Glu-437 was oriented to the hydroxyl group of the kaempferol and formed a strong hydrogen bond (bond length, 2.0 Å) interaction with kaempferol (Fig. 7D). Notably, the residue Tyr-469 had strong Van der Waals interactions with kaempferol because of the close proximity between each other (Fig. 7C). Regarding the other residues (i.e., Trp-435, Ile-468, Leu-470, Pro-471, and Ala-474), the majority of the decomposed energy interaction originated from Van der Waals interactions and apparent hydrophobic interactions. In addition, the total binding free energy calculated for the LLO-kaempferol complex revealed an ΔG*_bind_* (binding free energies) of approximately −13.3 kcal/mol, indicating that kaempferol could strongly bind to and interact with the predicted binding site of LLO (Fig. 7C).

In order to more definitively confirm the essential binding site based on the above-described results, we next introduced the mutations of LLO by replacing the predicted residues with Ala. By comparing the inhibition rate of kaempferol on the hemolytic activity of LLO_WT_ and involved mutants, we determined that the residues Glu-437, Tyr-469, and Ile-468 critically contributed to the LLO inhibition by kaempferol, as evident by the stronger inhibition of kaempferol on wild-type LLO (LLO_WT_) than those mutants (Fig. 7E). Consistently, the mutation of these essential binding sites also led to the invalidation of kaempferol in LLO-induced cytotoxicity (Fig. 7F), further confirming the interaction between kaempferol and LLO. Thus, these results verified that kaempferol blocked LLO pore formation mainly through engaging Glu-437, Tyr-469, and Ile-468 of LLO, which provided valuable information for further development of LLO inhibitors.

## DISCUSSION

Although most L. monocytogenes are currently susceptible to the antibiotics commonly used the in clinic, there is always a need to develop novel alternative approaches, because antibiotic resistance is considered to be a predicable result determined by the mechanism of action of antibiotics. The pore-forming toxin LLO appears to be a promising therapy target due to its indispensable role in L. monocytogenes pathogenesis and intracellular life cycle. Indeed, LLO also acts as a predominant antigen for T cells; thus, an LLO-targeted vaccine is also being developed to prevent listeriosis (28). In addition to vaccines and antibodies, chemical inhibitors also have great potential. The natural compound library represents an important resource for drug development, from which many lead compound have been excavated to combat bacterial infection through targeting key virulence or protein secretion systems. Kaempferol is a kind of natural flavonoid with abundant pharmacological activities, including antioxidant, antibiofilm, antiapoptosis, kinase-regulatory, and neuroprotective activities (29). So far, the potential use of kaempferol in the treatment of L. monocytogenes infection has not yet been explored. In the present study, we characterized kaempferol as a potential therapeutic for L. monocytogenes infection, which not only hindered L. monocytogenes pathogenesis by potently blocking LLO pore formation, but also inhibited the MyD88-dependent inflammation response.

Instead of targeting bacterial viability or toxin production, kaempferol engaged the residues Glu-437, Tyr-469, and Ile-468 of LLO, thereby efficiently resulting in the inactivation of LLO. Unlike the residues K175, S176, and E262, which play indispensable roles in the oligomerization and pore formation of LLO (30), all the binding sites determined in our study are not required for LLO pore formation. The pore complex formed by extracellular LLO is also critically required for bacterial entry in nonphagocytic cells (31), our results suggested that kaempferol might hinder L. monocytogenes internalization prior to intracellular replication. Surprisingly, our results suggested that kaempferol-driven inhibition on MAPK and NF-κB signaling was not fully mediated by LLO, but rather, that the small molecule simultaneously exhibited potent inhibition on the MyD88-dependent inflammation response downstream of TLR2/4. These results would enhance the knowledge of the anti-inflammation activity of kaempferol and broadened the applications of kaempferol in inflammatory diseases. Prior to the discovery that murine E-cadherins (Ecads) were not the host cellular receptors for InlA, this surface invasin was regarded as the key virulence factor mediating the translocation of L. monocytogenes from the intestinal lumen to other organs (32). However, accumulating evidence supports that LLO is also involved in the bacteria breaching the intestinal barrier during L. monocytogenes infection (6). Using an *in vitro* Caco-2 monolayer model, we demonstrated that kaempferol prevented LLO-induced barrier disruption, a critical hallmark of pathogenic L. monocytogenes infection, indicating that kaempferol might prevent L. monocytogenes from crossing host barriers. However, whether kaempferol reduced the bacterial load through this mechanism needs to be further explored in an oral infection model.

In fact, except for the investigations in the present study, the small molecule kaempferol also has some other significant merits in curbing L. monocytogenes infection. For example, autophagy is a protective mechanism for host defense against L. monocytogenes, where the bacterium is targeted for autophagic degradation at the early stage of infection (33), but a recent study found that kaempferol has the potential for autophagy (34), indicating that kaempferol might promote bacterial clearance through enhancing the autophagy pathway. Moreover, kaempferol was reported to stabilize the immunosuppressive function of FOXP3^+^ regulatory T cells (Tregs) by inhibiting PIM1, a protein kinase specifically catalyzing the phosphorylation of the FOXP3 (35). In this way, kaempferol may limit bacterial expansion during L. monocytogenes infection, because reduced Treg suppressive potency appears to eradicate infection via accelerating the expansion of protective antigen-specific CD8^+^ T cells in the case of acute L. monocytogenes infection (36). Last but not least, as opposed to the traditional broad-spectrum antibiotics that have a high risk of perturbing commensal bacteria in the gut, kaempferol was proved to rebalance the gut microbiota and microbial metabolism (37), which would preserve the first-line defense against numerous enteropathogenic bacterial infections, including L. monocytogenes. Here, we verified that kaempferol hardly influenced the viability of L. monocytogenes, suggesting a lower risk of inducing drug resistance than traditional antibiotics. The role of kaempferol in inhibiting LLO and inflammation provided new therapeutic indications for presenting this bioactive natural compound as an alternative approach to counteract L. monocytogenes infection.

In conclusion, our study discovered that, acting as a potent inhibitor of LLO pore formation and MyD88-dependent inflammation response, kaempferol effectively suppressed L. monocytogenes virulence and L. monocytogenes*-*evoked inflammation and thus exhibited good therapeutic efficiency against L. monocytogenes infection *in vivo*. These findings presented kaempferol as a promising therapeutic candidate for L. monocytogenes infection, which paved the load for the development of antibacterial infection strategies.

## MATERIALS AND METHODS

### Reagents.

Kaempferol (≥98%) was purchased from Herbpurify (Chengdu, China) and dissolved in dimethyl sulfoxide (DMSO; Sigma-Aldrich). A cytotoxicity detection kit (Roche, Switzerland) and Live/Dead cell imaging kit (Invitrogen, USA) were used for the analysis of cytotoxicity. T-PER tissue protein extraction reagents (Thermo Scientific, USA) and a bicinchoninic acid (BCA) protein assay kit (Beyotime, Shanghai) were used to prepare biological protein samples. Fluorescein sodium salt used for the evaluation of barrier permeability was purchased from Sigma-Aldrich. Total RNAiso reagent and a MutanBEST kit were from TaKaRa (Tokyo, Japan). FastStart Universal SYBR green master mix was purchased from Roche.

### Bacterial strains.

Escherichia coli DH5α and E. coli BL21 used for protein expression and purification were purchased from TransGen (Beijing, China) and cultured in LB medium. Wild-type L. monocytogenes strain EGD and its *hly*-deficient mutant EGDΔ*hly* and the complementation strain EGDΔ*hly*::*hly* were kind gifts from Pascale Cossart (Institut Pasteur, Paris, France) and were grown in Trypticase soy broth (TSB) medium with shaking at 37°C.

### Expression and purification of recombinant protein.

Recombinant LLO_WT_ and its mutants without signal peptide (amino acids 1 to 24) were heterologously overexpressed in E. coli BL21 based on the pET21a vector. In short, the overnight cultures were diluted 1:100 in the fresh LB medium containing 100 μg/mL ampicillin, and then protein expression was induced with 300 μM IPTG (isopropyl-β-d-1-thiogalactopyranoside) while reaching an optical density at 600 nm (OD_600_) of 0.6 to 0.8; at the same time, the culture temperature was turned down to 16°C. After 18 h, the bacterial pellets were obtained and sonicated for the collection of soluble protein fractions. The His-tagged proteins were purified using nickel-nitrilotriacetic acid (Ni-NTA) columns. After the concentrations were determined, the protein fractions were stored at −80°C.

### Hemolysis assay.

Erythrocyte leakage was determined by the release of hemoglobin that could be spectrophotometrically detected at 543 nm (38). Recombinant LLO proteins dispensed in the 96-well plates were incubated with the indicated concentrations of kaempferol at 37°C for 30 min before the addition of freshly washed sheep erythrocytes. The released hemoglobin was quantified after a 20-min incubation. The absorbance of samples treated with 0.1% Triton X-100 was regarded as the reference value for 100% lysis activity. Hemolysis was defined as the value of each sample relative to that of the 0.1% Triton X-100-treated sample.

### Specimen preparation and EM.

The specimen for electron microscopy (EM) was prepared as described previously (30). Briefly, the resealed ghosts were treated with 2 μg/mL LLO protein in the presence or absence of 32 μg/mL kaempferol and then transferred to EM grids, followed by negative staining with 1% uranyl acetate. Then, the specimens were analyzed with a Hitachi H-7650 transmission EM (Tokyo, Japan) operating at 80 kV.

### MIC determination and growth curve assay.

The MICs of kaempferol for the involved L. monocytogenes strains were determined by broth microdilution according to the guidance of the Clinical and Laboratory Standards Institute (CLSI) (39). To assess the effect of kaempferol on the viability of L. monocytogenes strain EGD, the growth curve assay was performed as previously described (40).

### Cell culture and treatments.

The human colon carcinoma cell line Caco-2 and mouse macrophage-like cell line J774A.1 were grown in Dulbecco’s modified Eagle’s medium (DMEM; Gibco) supplemented with 10% fetal bovine serum (FBS) and 100 U/mL penicillin G plus 100 μg/mL streptomycin sulfate. Mouse peritoneal macrophages (MPMs) derived from C57BL/6 mice were obtained as previously reported and cultured in RPMI medium with 10% FBS (41). All the cells were grown at 37°C in a 5% CO_2_ incubator. To investigate the effect of kaempferol on L. monocytogenes-caused inflammation response, MPMs grown overnight in the 6-well plate at 5 × 10^6^ cells/well were challenged with L. monocytogenes at a multiplicity of infection (MOI) of 5 for 5 h in the presence of dimethyl sulfoxide (DMSO) vehicle or 32 μg/mL kaempferol. To clarify the effect of kaempferol on the MyD88-dependent inflammation response downstream of TLRs, MPMs grown overnight in the 6-well plate at 5 × 10^6^ cells/well were stimulated with TLR2 ligand Pam3CSK4 (1 μg/mL) and TLR4 ligand LPS (1 μg/mL).

### Cytotoxicity assays.

Cell death was evaluated by the lactate dehydrogenase (LDH) release assay using a cytotoxicity detection kit (Roche). For the determination L. monocytogenes*-*caused cytotoxicity, J774 A.1 cells grown overnight in the 96-well plate at 1 × 10^5^ cells/well were challenged with L. monocytogenes at an MOI of 50. Meanwhile, the indicated concentrations of kaempferol were added into the coculture system. After a 6-h incubation at 37°C, cytotoxicity was determined by detecting the amount of LDH released in the supernatant according to the manufacturer’s instructions. Next, cells at the bottom were stained using a Live/Dead cell imaging kit, where the live cells and membrane-damaged dead cell were labeled with green and red fluorescence, respectively. Cells were visualized by an inverted fluorescence microscope (Olympus, Tokyo). To assess the effect of kaempferol on LLO-induced cytotoxicity, Caco-2 cells grown overnight in the 96-well plate at 2 × 10^4^ cells/well were stimulated with serially diluted LLO protein in the presence of DMSO vehicle or 32 μg/mL kaempferol. After a 3-h incubation at 37°C in 5% CO_2_, the cytotoxicity directly resulted from LLO stimulation was determined as described above.

### Immunoblot analysis.

Cells were washed with cold phosphate-buffered saline (PBS) and then lysed with T-PER mammalian protein extraction reagent. Cell debris was removed by centrifugation at 10,000 × *g* for 8 min (4°C). Then, the total protein concentrations of the supernatant fractions were determined using the BCA protein assay kit according to the manufacturer’s instructions, followed by the addition of 5× SDS loading buffer and heat denaturation at 100°C for 8 min. The protein samples were resolved in 5 to 10% SDS-PAGE gels and transferred to polyvinylidene difluoride membranes (Millipore). The target proteins on the membranes were probed with the specific primary antibodies as follows: rabbit polyclonal anti-JNK (Proteintech, catalog [cat.] no. 51151-1-AP), rabbit polyclonal anti-JNK phospho (Thr183 [221] plus Thr185 223) (Arigo, cat. no. ARG51807), rabbit monoclonal anti-p38 MAPK (CST, cat. no. 8690), rabbit monoclonal anti-p38 MAPK phospho (Thr180/Tyr182) (CST, cat. no. 4511S), rabbit polyclonal anti-ERK1/2 (Proteintech, cat. no. 16443-1-AP), rabbit polyclonal anti-ERK1/2 phospho (Thr202/Tyr204) (Arigo, cat. no. ARG52277), rabbit polyclonal anti-IKKα (Arigo, cat. no. ARG65746), rabbit polyclonal anti-IKKα phospho (Thr23) (Arigo, cat. no. ARG51630), rabbit monoclonal anti-NF-κB p65 (CST, cat. no. 8242), rabbit monoclonal anti-NF-κB p65 phospho (Ser536) (CST, cat. no. 3033S), mouse polyclonal anti-β-actin (Proteintech, cat. no. 66009-1-Ig), and horseradish peroxidase (HRP)-conjugated anti-rabbit (Proteintech, cat. no. SA00001-2) and anti-mouse IgG (Proteintech, cat. no. SA00001-1) secondary antibodies. All the primary antibodies and secondary antibodies were diluted 1:1,000 and 1:4,000, respectively, in Tris-buffered saline with Tween 20 (TBST). The immunoblots were developed with the enhanced chemiluminescence ECL kit (Biosharp) and visualized using an ECL Plus Western blotting detection system (Tanon). The Western blot bands were semiquantitated using Image-Pro software.

### Measurement of cytokines.

The levels of cytokines TNF-α, IL-6, and IL-1β in the culture supernatants as well as liver and spleen homogenates were measured using the mouse enzyme-linked immunosorbent (ELISA) MAX Deluxe set (BioLegend) according to the manufacturer’s instructions.

### RNA isolation and RT-qPCR.

Total RNA was isolated using RNAiso reagent and then reverse-transcribed into cDNAs using a Prime Script reverse transcription (RT) reagent kit (Tokyo, Japan). Reverse transcription-quantitative PCR (RT-qPCR) was performed using FastStart Universal SYBR green master mix, and the gene-specific primer pairs are listed in Table 1. Samples were loaded onto 96-well reaction plates and run on an Applied Bioscience 7500 thermocycler. Data were analyzed by the ΔΔ*CT* method and normalized to GAPDH abundance. The data shown in the graph represent the fold induction relative to untreated cells.

**TABLE 1:** Sequence of primers used for RT-qPCR

Gene	Primer	Sequence (5′–3′)
Mouse GAPDH	Sense	AGGTCGGTGTGAACGGATTTG
	Antisense	GGGGTCGTTGATGGCAACA
Mouse IL-1β	Sense	ACCTGTGTCTTTCCCGTGG
	Antisense	TCATCTCGGAGCCTGTAGTG
Mouse IL-6	Sense	CCACTTCACAAGTCGGAGGCTTA
	Antisense	GCAAGTGCATCATCGTTGTTCATAC
Mouse TNF-α	Sense	CCTATGTCTCAGCCTCTTCTCAT
	Antisense	CACTTGGTGGTTTGCTACGA

### Measurements of transepithelial electrical resistance (TEER) and permeability of the Caco-2 monolayer.

Caco-2 cells were grown in Corning Transwell permeable supports (0.4 μM) to mimic the intestinal epithelial barrier *in vitro* (42). After 14 days of differentiation, the monolayer resistance was documented every 3 days using an epithelial voltohmmeter (Millicell ERS-2) until approximately 600 to 800 Ω was reached. On the day of experiment, the monolayer was washed with warm PBS and the culture medium with replaced with serum-free DMEM. Then, sodium fluorescein was added to the insert compartment at a final concentration of 0.01 mg/mL, followed by the addition of LLO protein (1 μg/mL) treated with DMSO vehicle or 32 μg/mL kaempferol. TEER was measured for the first 10 min and then at intervals of 30 min, and at the same time, 100 μL of medium in the basal chamber was moved to the 96-well plate to quantify the percolated fluorescein by measuring the fluorescence intensity at 520 nm with an excitation of 460 nm. The TEER changes were presented as the mean percentage of the initial TEER values.

### Drug administration and mouse infection.

C57BL/6 female mice (18 to 20 g) and BALB/c female mice aged 6 to 8 weeks (20 to 25 g) were purchased from Liaoning Changsheng Biotechnology Co. Ltd. (Ben Xi, China) and maintained in individually ventilated cages (IVCs) under specific-pathogen-free and standard housing conditions (25 ± 1°C, 55% humidity, 12-h light/dark cycle) with free access to standard pellet food and sterilized water. All the *in vivo* experiments were carried out with the approval of the Jilin University Institutional Animal Care Committee and strictly conducted in accordance with the guidelines.

For animal injection, kaempferol stock initially dissolved in DMSO (100 mg/mL) was diluted to 40 mg/mL with sterilized water containing 5% polyethylene glycol (PEG) 400 and 5% Tween 80. To perform the survival rate analysis, mice were randomly divided into a vehicle group and a kaempferol group (*n* = 13 mice each group). Mice in the vehicle group were intraperitoneally inoculated with 5 × 10^6^ CFU of wild-type L. monocytogenes EGD and subcutaneously administered with 50 μL vehicle (sterilized water containing 5% PEG 400 and 5% Tween 80). Mice in the kaempferol group were challenged with same amount of EGD and treated with 100 mg/kg kaempferol. Following infection, drug delivery was maintained at intervals of 8 h for 4 days, and the survival rate of the mice was recorded for 6 days. Finally, the mice that survived were anaesthetized with 5% isoflurane by inhalation and then killed by rapid cervical dislocation.

To further assess bacterial burden and cytokine levels in livers and spleens, as well as the histopathology of the two target organs, a negative group was created (*n* = 7 mice in each group). In the experiment, mice in the negative group received only vehicle, mice in the vehicle group were intraperitoneally challenged with 5 × 10^6^ CFU of wild-type L. monocytogenes EGD and received 50 μL vehicle, and mice in the kaempferol group were challenged with the same amount of EGD and administered with 100 mg/kg kaempferol at intervals of 8 h following infection. Then, 48 h after infection, mice were anaesthetized with 5% isoflurane and then killed by rapid cervical dislocation. The liver and spleen of each mouse were dissociated and homogenized in sterilized PBS (10% [wt/vol]). Bacterial burden was determined by microbiological plating, and cytokine levels in the supernatants of those tissue homogenates were measured using a mouse ELISA kit according to the manufacturer’s instructions. For histopathological analysis, the spleens and livers were fixed with 10% formalin and stained with hematoxylin and eosin (H&E). The pathological sores of liver and spleen were evaluated as previously described (43).

### Molecular docking and dynamics analysis.

The kaempferol molecule docking with the three-dimensional (3D) structure of the LLO (PDB ID: 4CDB) was performed using AutoDock Vina 1.1.2 and ChemBioDraw Ultra 14.0. Then, the molecular dynamics was performed on a Dell Precision T5500 workstation, and the Amber 14 and AmberTools 15 programs were used for molecular dynamics simulations of the selected docked pose. To further explore the key protein residues responsible for the binding mode, the binding free energy was decomposed on a per-residue basis.

### Site-directed mutagenesis.

Site-directed mutants of LLO were all generated using the MutanBEST kit based on the DNA template of a wild-type construct. The primer pairs used for mutations are shown in Table 2. Finally, the sequences of all mutant constructs were verified by nucleotide sequencing.

**TABLE 2:** Sequence of primers used for site-directed mutagenesis

Gene	Primer	Sequence (5′–3′)
LLO_K93A_	Sense	CCGCCAAGAAAAGGTTACGCCGATGGAAATGAATATATTG
	Antisense	CAATATATTCATTTCCATCGGCGTAACCTTTTCTTGGCGG
LLO_W435A_	Sense	GTTGCTCAATTCAACATTTCTGCGGATGAAGTAAATTATGATCC
	Antisense	GGATCATAATTTACTTCATCCGCAGAAATGTTGAATTGAGCAAC
LLO_E437A_	Sense	CATTTCTTGGGATGCGGTAAATTATGATCC
	Antisense	GGATCATAATTTACCGCATCCCAAGAAATG
LLO_K451A_	Sense	GAAATTGTTCAACATGCGAACTGGAGCGAAAAC
	Antisense	GTTTTCGCTCCAGTTCGCATGTTGAACAATTTC
LLO_I468A_	Sense	CATTTCACATCGTCCGCTTATTTGCCTGGTAAC
	Antisense	GTTACCAGGCAAATAAGCGGACGATGTGAAATG
LLO_Y469A_	Sense	CACATCGTCCATCGCGTTGCCTGGTAAC
	Antisense	GTTACCAGGCAACGCGATGGACGATGTG
LLO_L470A_	Sense	CATCGTCCATCTATGCGCCTGGTAACGCG
	Antisense	CGCGTTACCAGGCGCATAGATGGACGATG
LLO_P471A_	Sense	GTCCATCTATTTGGCTGGTAACGCGAG
	Antisense	CTCGCGTTACCAGCCAAATAGATGGAC

### Statistical analysis.

The numeric data are shown as the mean ± the standard error of the mean (SEM), and the statistical analyses were performed using GraphPad Prism 5.0 software. The comparison between two independent treatment groups was carried out with Student’s unpaired two-tailed *t* test. Multiple analyses between more than two groups were performed by one-way analysis of variance (ANOVA) with Bonferroni’s correction. Mouse survival curves and the statistics were presented and analyzed using the Mantel-Cox log-rank test. *, *P < *0.05; **, *P *< 0.01; *P < *0.05 was considered to be statistically significant.
